# Focus on the role of mixed micelles and lipid droplets in the oxidative stability of oil-in-water emulsions using size distribution Taylor dispersion analysis

**DOI:** 10.1016/j.crfs.2026.101369

**Published:** 2026-02-28

**Authors:** Erwann Durand, Théo Troncho, Hiteshree Koli, Camille Robichon, Pierre Villeneuve

**Affiliations:** a CIRAD, UMR QualiSud, Montpellier, F-34398, France; b QUALISUD, Univ Montpellier, Avignon Université, CIRAD, Institut Agro, IRD, Université de La Réunion, Montpellier, France

**Keywords:** Lipids, Oxidation, Micelles, Tween, Emulsion, Size distribution, Taylor dispersion analysis

## Abstract

This study investigates the role of mixed micelles and lipid droplets in the oxidative stability of oil-in-water emulsions using Size Distribution - Taylor Dispersion Analysis. Tween surfactant facilitates the formation of mixed micelles containing surface-active components in non-stripped oils, whereas oil stripping suppresses their formation, indicating that these micelles are enriched in non-TAG-derived species such as DAG, MAG, and FFA. Tween 20, and to a lesser extent Tween 80, also promotes the incorporation of TAG molecules into mixed micelles, which can reorganize into small lipid droplets (<100 nm) under low-energy conditions. The fraction of oil solubilized in Tween 20 showed minimal dependence on homogenization pressure or number of passes, confirming that mixed micelles readily form under mild energy inputs. In contrast, the distribution of oil between small and larger droplets (>500 nm) is strongly influenced by emulsification energy. During oxidation, emulsions with narrow droplet-size distributions retain both their small droplet size and the proportion of non-oxidized oil within each population. In contrast, polydisperse systems exhibit an increased amount of non-oxidized oil in small droplets relative to mixed micelles, accompanied by droplet growth. These results highlight how surfactant type, mixed-micelle structure, droplet size distribution, and emulsification energy collectively govern oil partitioning and oxidative behavior in emulsions.

## Highlights

• SD-TDA resolves mixed micelles and lipid droplets and quantifies oil partitioning in O/W emulsions.
• Tween 20 forms mixed micelles enriched in surface-active lipids, largely independent of homogenization energy.
• Emulsification energy controls droplet size distribution and droplet-to-micelle oil ratios.
• Mixed micelles and small droplets seem to exhibit comparable oxidation rates.
• Large lipid droplets act as slow-oxidizing TAG reservoirs, modulating oxidation pathways in polydisperse systems.

## Graphical abstract

**Figure:**
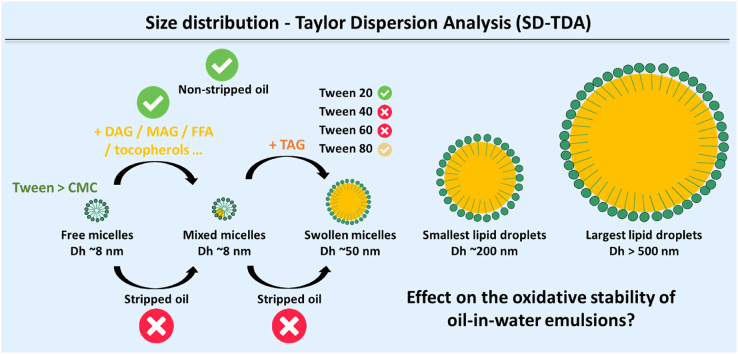

## 1 Introduction

Lipid oxidation is the primary cause of deterioration in foods and significantly influences the sensory qualities, nutritional value, and shelf-life of foods rich in lipids. Controlling this process is therefore crucial, especially in products high in (poly)unsaturated fatty acids, which are particularly prone to oxidation (Schaich, 2020). Although lipid oxidation has been widely investigated in bulk oils, most dietary lipids are actually consumed in emulsified forms, particularly oil-in-water (O/W) emulsions. These emulsions consist of oil droplets dispersed in water, stabilized by surface-active molecules at the oil-water interface. This interfacial region is known to play a crucial role as the site where lipids, oxygen, and pro-oxidants interact. In addition, smaller droplets, due to their greater specific oil–water interfacial area, are generally expected to undergo more rapid lipid oxidation. Although this tendency has often been confirmed experimentally, conflicting findings have also been reported. These inconsistencies likely stem from the fact that variations in droplet size are frequently accompanied by simultaneous changes in other emulsion characteristics, such as interfacial composition, surfactant coverage, emulsification energy input, and the distribution of pro-oxidants and antioxidants (McClements and Decker, 2000; Berton-Carabin et al., 2014; Villeneuve et al., 2021; Hennebelle et al., 2024). Consequently, some studies observe accelerated oxidation in smaller droplets, while others report greater susceptibility in larger ones. Thus, multiple factors, including droplet size, interfacial composition, emulsifier type, and the behavior of pro- and antioxidant species affect lipid oxidation in emulsified systems, making the process complex and challenging to predict or control (McClements and Decker, 2000; Berton-Carabin et al., 2014; Villeneuve et al., 2021; Hennebelle et al., 2024). In oil-in-water (O/W) emulsions, food emulsifiers can be classified into three main groups: (i) low-molecular-weight emulsifiers (LMWEs), also known as surfactants, which typically contain a polar or charged hydrophilic headgroup and one or more hydrophobic alkyl chains (Hasenhuettl and Hartel, 2008; McClements, 2015); (ii) amphiphilic biopolymers, which are larger molecules with hydrophilic and hydrophobic segments arranged in either regular or random patterns with proteins being a key example of this group, and (iii) colloidal particles that possess surface characteristics allowing partial wettability by both oil and water, enabling strong adsorption at the oil–water interface and the formation of so-called Pickering emulsions (Dickinson, 2020; Berton-Carabin and Schroën, 2015). An important aspect to note about emulsifiers is that food emulsions are frequently prepared with a much higher amount of emulsifier than what is strictly necessary to coat and stabilize the oil–water interface (Berton-Carabin et al., 2014). As a result, only a small portion of the emulsifier participates in stabilizing the oil droplets, while the majority remains dispersed in the continuous phase. In the case of low-molecular-weight surfactants, this emulsifier surplus portion may exist not only as dissolved monomeric molecules but can also organize into different colloidal structures, such as micelles when the surfactant concentration exceeds the critical micelle concentration (CMC) (Berton-Carabin and Villeneuve, 2023). These micelles are not inert regarding lipid oxidation pathways but play an important role in mass transport phenomena that strongly influence lipid oxidation kinetics. Indeed, when surfactant micelles are present in the continuous phase, they can alter the distribution of various components involved in lipid oxidation processes (Villeneuve et al., 2021; Hennebelle et al., 2024). For example, micelles are capable of binding metal ions (Richards et al., 2002) or solubilizing lipophilic antioxidants such as tocopherols (Kiralan et al., 2014; Inchingolo et al., 2021). Moreover, it has been proposed that surfactant micelles may facilitate the physical propagation of oxidation within emulsions by transporting lipid oxidation intermediates through the continuous phase (ten Klooster et al., 2023a; Laguerre et al., 2020). Regarding interactions of surfactants micelles with antioxidants present in the emulsions, we recently used Size Distribution - Taylor Dispersion Analysis (SD-TDA) to evaluate the incorporation of α-tocopherol, curcumin, gallic acid and gallate esters into sodium dodecyl sulphate (SDS) micelles. SD-TDA is a technique used to determine the diffusion coefficients and hydrodynamic radii of molecules. This method is based on the dispersion of a solute plug traveling through a uniform cylindrical tube under laminar Poiseuille flow. By applying the principles of Taylor dispersion and using absorbance detection to monitor the solute's movement through a capillary, this method allows for precise measurement of diffusion coefficients and particle sizes in solution (Durand et al., 2025). Consequently, SD-TDA is suitable for analytes of a wide size range, from angstrom-scale to submicrometer, and has proven to be an effective approach for characterizing the size of diverse nanoscale entities, including single molecules, peptide and protein aggregates (Norrild et al., 2024; Roca et al., 2024), liposomes (Franzen et al., 2011), or extracellular vesicles (Obeid et al., 2023).

In the present study, we again used SD-TDA to evaluate the impact of oil-in-water emulsion processing on the distribution of oil between micelles, small lipid droplet and large ones, and the impact of such distribution on the oxidative stability of the emulsion. In industrial applications, emulsification is typically carried out using high-pressure homogenizers or rotor–stator systems, with the required number of passes being influenced by the extent of droplet recoalescence, and vice versa (Berton-Carabin et al., 2018). Emulsification conditions, such as the equipment used and the amount of energy applied, are crucial, as they determine the resulting droplet size distribution, which in turn affects the emulsion's oxidative stability by altering the total surface area (Hennebelle et al., 2024; Neves et al., 2017; Walker et al., 2015). In theory, smaller droplets, with their increased oil–water interfacial area should accelerate lipid oxidation (Berton-Carabin et al., 2014). Although this trend has frequently been observed experimentally, conflicting findings exist, likely due to the difficulty of modifying droplet size without simultaneously altering other emulsion characteristics. Some studies observed that smaller droplets oxidize faster (Li et al., 2020; Gohtani et al., 1999; Jacobsen et al., 2000; Lethuaut et al., 2002; Rampon et al., 2001; Kuhn and Cunha, 2012; Ma et al., 2013; Azuma et al., 2009; Yang et al., 2020) whereas other observed the opposite with larger droplet being more sensitive to oxidation (O'Dwyer et al., 2013; Ries et al., 2010; Nakaya et al., 2005; Sørensen et al., 2007; Atarés et al., 2012; Costa et al., 2020). In a recent study, ten Klooster et al. (ten Klooster et al., 2023a) investigated the occurrence and effect on oxidative stability of very small oil droplets and swollen micelles in surfactant-stabilized emulsions, and found that increasing the mechanical forces used to make the emulsion increased the amount of oil in the very small droplets. These authors also observed that lipid oxidation products were overrepresented in the very small droplets and hypothesized that the large surface-to-volume ratio of smaller droplets compared to the larger droplets favors the contact between traces of pro-oxidants metal ions present in the continuous phase. In this present work we used the capacity of SD-TDA to detect very small objects such a micelles, swollen micelles and small lipid droplets to further investigate the findings by ten Klooster et al. (ten Klooster et al., 2023a) and try to evaluate the impact of oil distribution within these different objects on oxidative stability of the emulsion. Tween surfactants were chosen to stabilize the oil-in-water emulsions in this study because they are widely used low-molecular-weight emulsifiers and are representative of those commonly employed in investigations of lipid oxidation in emulsion systems.

## 2 Materiel and method

### 2.1 Chemicals

Lipid standards, including phosphatidylcholine (PC), phosphatidylserine (PS), phosphatidylinositol (PI), phospahtidylethanolamine (PE), phosphatidylglycerol (PG) were purchased from Avanti Polar Lipids, Inc. (Alabaster, AL, USA). The fatty acid methyl ester (FAME) standard mix (37-component FAME Mix), was acquired from Supelco (Darmstadt, Germany). 3-Phosphatidic acid (PA) was obtained from Thermo Fischer Scientific (Waltham, MA, USA). Copper (II) sulphate, and phosphoric acid (≥85%), acetic acid (≥99.8%), diethylether, and methanol (≥99.0%) were obtained from Honeywell Fluka (Seelze, Germany). Aluminum oxide was purchased from Merck (Darmstadt, Germany). Triolein, oleic acid, sodium methylate, acetyl chloride (≥99.0%), phosphate buffer solution (PBS pH 7.2), Tween 20, Tween 40, Tween 60, Tween 80 and chloroform (≥99.8%) were purchased from Sigma-Aldrich (Saint-Quentin-Fallavier, France). Phenolphthalein and hexane (≥99.0%) were purchased from Carlo Erba (Val-de-Reuil, France), and tung oil (average MW = 872 g/mol) was purchased from Furniture Clinic NL (Wijngaardsweg, Heerlen, Nederland).

### 2.2 Determination of Total Fatty Acid (TFA) profiles of tung oil

The TFA profiles of tung oil were determined by gas chromatography (GC) according to the NF T60-233 standard method. Briefly, sodium methylate solution (1 mL) with phenolphthalein was added to oil sample (15 mg). After heating at 65 °C for 10 min, hydrochloric methanol (1 mL) was added. The mixture was again heated at 65 °C for 10 min. After cooling, hexane (1 mL) and water (1 mL) were added. The mixture was centrifuged at 1500 rpm for 5 min using a Hettich Rotina 380R (Hettich, Tuttlingen, Germany), and the organic phase was collected. A 1 μL aliquot of the organic layer was then injected into the GC system. GC analyzes were performed with a Focus GC (Thermo Electron Corporation, Massachusetts, USA), equipped with a split injector (ratio of 1/20), CP-Cil 88 Varian capillary column (50 m × 0.25 mm with 0.2 μm film thickness; Agilent Chrompack), and helium (1 mL min^−1^) as carrier gas. Fatty acid methyl esters (FAME) were analyzed by a flame ionization detector and ChromCard software data system (version 2005, Thermo FisherScientific, Massachusetts, USA). The column temperature started from 150 °C, reached 225 °C with a rise of 5 °C.min^−1^ and was maintained at 225 °C for 10 min. The injector and detector temperatures were 250 and 270 °C, respectively. FAME were identified using the retention time of external standards of FAME mixture (mix37 EMAG Supelco). (C16:0, 2.3%; C18:0, 2.2%; C18:1n9c, 5.8%; C18:1n7, 0.3%; C18:2n6c, 6.9%; C20:0, 0.2%; C18:3 (linoleic acid), 0.7%; C18:3 (α-eleostearic acid), 78.7%; C18:3 (β-eleostearic acid), 1.9%).

### 2.3 Preparation of oil-loaded in Tween micelles

Tween micelles (Tween 20, Tween 40, Tween 60 or Tween 80) were prepared at a concentration of 1% (w/w) in 20 mL of phosphate buffer (pH 7.2). Then, 1% (w/w) of tung oil (200 mg) was added to each Tween micelle solution. The headspace of each vial was purged with nitrogen gas to remove oxygen, the caps were sealed, and the samples were incubated at 30 °C under vigorous orbital stirring (300 rpm) for 4 h and 24 h using an IKA KS 4000 i-control incubator (IKA, Staufen, Germany). After incubation, 3 mL of each Tween micelle preparation was collected and centrifuged at 5000 rpm for 5 min (Rotina 380R, Hettich, Westphalia, Germany) to physically separate the top oil phase from the bottom dispersed phase in buffer. 1.5 mL of the bottom dispersed phase in buffer was collected and transferred into a Size Distribution - Taylor Dispersion Analysis **(**SD-TDA) vial for analyses.

### 2.4 Extraction of lipids and Thin-Layer Chromatography (TLC) analysis

Lipids were extracted from both top oil phase and the bottom dispersed phase in buffer using a modified Folch method. Briefly, a 5 ml sample aliquot was combined with 15 ml of Folch solvent (Chloroform/Methanol, 2:1 v/v) and vortexed for 30 s. Phase separation was induced by adding 5 ml of MilliQ water and vortexing for another 30 s. The resulting mixture was then centrifuged at 3500×*g* for 5 min (Rotina 380R, Hettich, Westphalia, Germany). The bottom chloroform phase, containing the extracted lipids, was carefully recovered and dried completely under a stream of nitrogen gas. The isolated lipids were subsequently resolubilized in 1.5 ml of chloroform for subsequent analysis. The resolubilized lipid extract (5 μl) and relevant lipid standards (Triacylglycerols (TAG), Diacylglycerols (DAG), Monoacylglycerols (MAG), and Free Fatty Acids (FFA)) were applied to HPTLC silica gel 60 pre-coated plates (Merck) using a CAMAG ATS4 apparatus. Lipid separation was achieved using a hexane/diethyl ether/acetic acid (70:30:1 v/v/v) solvent system. The migration was conducted in a CAMAG ADC2 apparatus under the following controlled parameters: Saturation time for 3 min, plate pre-conditioning time for 1 min, migration distance of 80 mm, and drying time for 3 min. Following migration, lipids were visualized by dipping the plates in an aqueous copper sulphate/85% phosphoric acid (50/50 v/v) solution, followed by drying and heating at 100 °C for 10 min to visualize the lipid bands.

### 2.5 Tung-oil-in-water emulsion preparation

The oil-in-water emulsion (500 mL) was prepared using 1% (w/w) tung oil and 1% (w/w) Tween 20 in phosphate-buffered aqueous solution (PBS, pH 7.2). The mixture was first pre-emulsified using a IKA T25 digital Ultra-Turrax homogenizer at 7600 rpm for 3 min (Janke & Kunkel, Staufen, Germany), followed by high-pressure homogenization with a LM10 Microfluidizer (Microfluidics, Westwood, USA) equipped with an F12Y chamber. Homogenization was performed at varying pressures (200, 350 and 500 bars) with either 2 or 5 passes. The chamber temperature was maintained at 10 °C using a water bath to prevent overheating during high-shear processing.

### 2.6 Size Distribution - Taylor Dispersion Analysis (SD-TDA)

The particle size distribution of samples was measured by Size Distribution – Taylor Dispersion Analysis (SD-TDA) using TaylorSizer (Nanoscale Metrix, France). Tween solution (1%wt) in phosphate buffer (pH 7.2) was used for both conditioning and mobilization steps. The capillary (fused silica tubing), measuring 60 cm with 49.5 cm effective length and an internal diameter of 50 μm, was lowered into the vial for sample injection and mobilization. It was initially conditioned with the Tween-buffer solution at 1000 mbar for 150 s. The sample was then injected into the capillary at 25 mbar for 18 s ensuring that the injection is equivalent to 1% of the total volume of the capillary. Subsequently, the Tween-buffer solution was used for sample mobilization at pressures of 50 mbar for durations of 1350 s. This process allowed the sample to migrate through the capillary until detection by the UV detector, which was set to wavelengths corresponding to the maximum absorbance of non-oxidized (273 nm) and oxidized (234 nm) Tung oil (Laguerre et al., 2008). All experiments were performed at 15 °C. The resulting peak was analyzed to determine the size distribution using TaylorSizer software (Taylorsoft) by setting the maximum size for data processing and analysis at 500 nm (Cottet et al., 2012).

### 2.7 Calibration curve of tung oil-in-water emulsions using varying droplet concentrations

First, a 1L tung oil-in-water emulsion was pre-emulsified following the protocol described in section 2.2. and then divided into four samples (250 mL each) which were subsequently subjected to high-pressure homogenization using a LM10 Microfluidizer (Microfluidics, Westwood, USA) equipped with an F12Y chamber at varying pressures 500, 600, 700, and 800 bar, each for five passes. Then, 1 mL of each emulsion was analyzed (n = 3) by Taylor Dispersion Analysis (TDA) to determine the integrated area of the oil signal from the Taylorgram. The objective was to identify the pressure condition at which a plateau in the integrated area was reached, indicating maximal dispersion of the 1% (w/w) Tung oil droplets. Based on this screening, the condition that yielded the plateau (700 bar, 5 passes) was selected and sequentially diluted in PBS (pH 7.2) containing 1% (w/w) Tween 20 to obtain a calibration range from 0 to 1% (w/w) Tung oil (0, 0.01%, 0.05%, 0.1%, 0.2%, 0.4%, 0.6%, 0.8%, 1%). UV absorbance of the emulsions at 273 nm and 234 nm were measured to construct a calibration curve for Tung oil concentration. The calibration curve exhibited a linear relationship (y = ax + b), consistent with the Beer–Lambert law. This calibration was used to determine the actual Tung oil content in emulsions prepared under different homogenization conditions by analyzing their corresponding Taylorgrams using TDA.

### 2.8 Quantification of tung oil loading into nano-objects and oxidation assessment via Taylorgram area integration

The quantification of Tung oil loading into various nano-objects and the evaluation of its oxidative state were performed using Size Distribution–Taylor Dispersion Analysis (SD-TDA) with a UV-TaylorSizer instrument (Nanoscale Metrix, France). Measurements were carried out at 273 nm to quantify the presence of (non-oxidized) Tung oil, and at 234 nm to monitor the formation of conjugated dienes, indicative of lipid oxidation. The amount of encapsulated Tung oil in each nano-object formulation (as determined in section 2.6) was quantified by comparing the integrated areas under the experimental Taylorgrams with the theoretical values derived from the calibration curve established in section 2.7.

The extent of oxidation was assessed by calculating the ratio of absorbance (or integrated area) at 234 nm to that at 273 nm, providing an estimate of the relative level of lipid oxidation in the emulsified systems, as follow:

$$Oxidation=\left(\frac{\frac{A_{273}\left(t\right)}{A_{234}\left(t\right)}-\frac{A_{273}\left(t\infty \right)}{A_{234}\left(t\infty \right)}}{\frac{A_{273}\left(t0\right)}{A_{234}\left(t0\right)}-\frac{A_{273}\left(t\infty \right)}{A_{234}\left(t\infty \right)}}\right)\times 100$$

$$Oxidation=\left(\frac{R\left(t\right)-R\left(t\infty \right)}{R\left(t0\right)-R\left(t\infty \right)}\right)\times 100$$

A_273_(t): area under the peak at 273 nm at time t.

A_234_(t): area under the peak at 234 nm at time t.

A_273_(t∞)/A_234_(t∞): values at boundary conditions (end of oxidation).

A_273_(t0)/A_234_(t0): initial values (time 0).

### 2.9 Dynamic Light Scattering (DLS)

The particle size distribution of samples was measured by Dynamic Light Scattering (DLS) using a Zetasizer pro (Malvern Panalytical, UK). A 20 μL emulsion sample was diluted in 1.08 mL of Milli-Q water and placed in a polystyrene cuvette (Fisherbrand, Fisher Scientific, France). The cuvette was inserted into the measurement chamber and a 1-min stabilization period was allowed prior to measurement. The refractive index was set to 1.47 for Tung oil and 1.33 for the dispersant (PBS). Measurements were performed at 25 °C using the general-purpose analysis model, in triplicate, and measures (n = 4) were reported as Z-average size (nm) and polydispersity index (PDI), as well as D(1,0) ± SD, D(3,2) ± SD and D(4,3) ± SD.

### 2.10 Statistical analyses

Each sample was prepared in triplicate and measurements were performed three times for each triplicate. Results are expressed as mean ± SD. Statistical significance was assessed by Student *t*-test (XLSTAT software). Values marked with an asterisk were considered significantly different (p < 0.05).

## 3 Results and discussion

### 3.1 Effect of Tween surfactant type on micelle size and oil loading

**Fig. 1:**
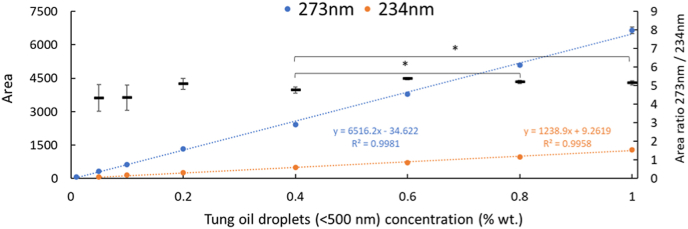
Total integration area (at 273 nm and 234 nm) of the Taylorgram peak of tung oil in-in-water prepared with varying tung oil concentrations (0.01–1 wt.%), and 1% (w/w) Tween 20 in phosphate-buffered solution (PBS, pH 7.2). Values marked with an asterisk∗ were considered significantly different (p < 0.05, n = 3).

Size distribution by Taylor dispersion analysis (SD-TDA) enables the determination of hydrodynamic diameters for UV-absorbing molecules within complex systems, while preserving the native structural state of the medium. To investigate these parameters in oil-in-water emulsions, tung oil was selected owing to its intrinsic property of exhibiting strong and selective absorbance at 273 nm, attributed to its high content (∼70%) of conjugated α-eleostearic acid. Considering that the size of nano-objects for particles smaller than 500 nm does not significantly affect the absorbance coefficient, a calibration curve of tung oil dispersed in 1 wt% Tween 20 in PBS solution (pH 7.2) was drawn up, following the procedure described in section 2.7 (Fig. 1). Under these conditions, SD-TDA enabled us to qualitatively identify and size the different very small populations (e.g., micelles ∼10 nm vs. small droplets ∼100 nm), as well as to quantitatively estimate the mass of α-eleostearic-acid-derived tung oil molecules in each population.

To evaluate the effect of Tween surfactant type on micelle sizes and oil loading, different Tween surfactants – namely Tween 20 (Tw20), Tween 40 (Tw40), Tween 60 (Tw60) and Tween (Tw80) – varying by the length of their hydrophobic alkyl chain (from C12 to C18 carbons, with an additional unsaturation in the case of Tw80), were evaluated for their ability to encapsulate tung oil within their micelles. This set of experiments was performed in the absence of high-pressure energy inputs for homogenization (see 2.3).

**Fig. 2:**
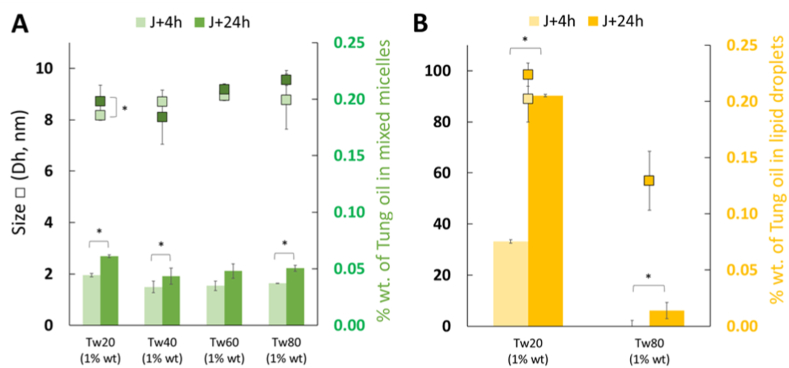
A/Size and oil content distributed in micelles (mixed micelles) formed by Tween 20 (Tw20), Tween 40 (Tw40), Tween 60 (Tw60) and Tween 80 (Tw80) added at 1%wt in PBS buffer pH 7.2, after 4 h and 24 h of incubation. B/Size and oil content distributed in small lipid droplets formed by Tween 20 (Tw20), Tween 40 (Tw40), Tween 60 (Tw60) and Tween 80 (Tw80) added at 1%wt in PBS buffer pH 7.2, after 4 h and 24 h of incubation. Note: For instance, 0.05 wt% oil is equivalent to 5% of the initial oil content. Values marked with an asterisk∗ were considered significantly different (p < 0.05, n = 3).

As shown in Fig. 2A, all Tween surfactants (1 wt%) were capable of solubilizing tung oil (initially added at 1 wt%), with concentrations ranging from 0.034 ± 0.005 wt% for Tw40 to 0.044 ± 0.002 wt% for Tw20. A slight effect of incubation time (4 h vs 24 h) was observed, with oil incorporation increasing after 24h to values ranging from 0.043 ± 0.004 wt% for Tw40 to 0.061 ± 0.001 wt% for Tw20. The hydrodynamic diameter (Dh) of oil-loaded micelles remained relatively constant across the different surfactants, showing only a slight increase, with sizes ranging from 8.44 ± 0.39 nm for Tw20 to 9.16 ± 0.85 nm for Tw80. For comparison, the size of unloaded Tw80 micelles, determined under the same conditions by SD-TDA with UV detection at 205 nm, was 8.83 ± 0.20 nm, which fall within the range of estimated sizes from previous studies (Luschtinetz and Dosche, 2009; Vafakish and Wilson, 2021; Karjiban et al., 2012). Under these mild incubation conditions, small oil droplets were also detected by SD-TDA, particularly with Tw20 and Tw80 (Fig. 2B). For Tw20, droplets of approximately 88.96 ± 9.01 nm containing 0.075 ± 0.001 wt% tung oil were consistently observed after 4 h, and their size and oil content significantly increased over time, reaching 98.51 ± 4.52 nm with 0.205 ± 0.004 wt% tung oil after 24 h. In the case of Tw80, droplets of about 56.87 ± 11.49 nm containing 0.014 ± 0.002 wt% tung oil were detected after 24 h of incubation. With Tw40 and Tw60, we did not observe the formation of such tiny droplets. Instead, inconsistent results were obtained, with occasional detection of droplets (>200 nm) that contained only a very small amount of integrated oil (<0.01 wt%). The formation of mixed micelles with Tw20 (and to a lesser extent Tw80) appears to dynamically alter the structure and integrity of the micelles, through mechanisms that would lead to a mass gain caused by micelle swelling via interfacial permeabilization loading TAG molecules and/or mixed-micelle fusion (Quinn et al., 2022; Knoch et al., 2021). Since Tw20 yielded the most interesting results, additional experiments were performed, but using stripped tung oil in this case. Interestingly, with stripped oil, that correspond to an oil fully depleted of its most surface-active compounds (monoacylglycerols (MAG), Diacylglycerols (DAG), free fatty acids (FFA), tocopherols, sterols, etc., see Fig. S1), markedly different results were observed. No mixed micelles containing tung oil fractions were detected (below the sensitivity limit of <0.004 wt%, corresponding to <0.4% of the 1 wt% oil initially added). Moreover, only trace amounts of oil small droplets ranging from ∼70 to ∼120 nm was observed, reaching 0.022 ± 0.003 wt% tung oil after 24 h, which is an order of magnitude lower than the 0.205 ± 0.004 wt% measured with non-stripped oil. These findings confirm that oil stripping may significantly affect the ability to form mixed micelles and could explain why TAG-bound hydroperoxides did not transfer from one lipid droplet to another in emulsions systems (ten Klooster et al., 2023b). This results also suggest that with non-stripped oil, mixed micelles essentially contain integrated surface-active lipids (polar lipids, DAG, MAG, FFA, tocopherols, etc.), which would then transition into swollen micelles and very small lipid droplets by loading TAG (or TAG-bound hydroperoxides) molecules (Nose and Numasawa, 2001; Wang et al., 2017; Totland and Blokhus, 2017; Bochynek et al., 2023). To verify this assumption, we have separated the non-loaded oil from the dispersed phase by centrifugation and analyzed their lipid composition (Fig. S1). The results confirmed that, in the case of non-stripped oil, the dispersed phase contained noticeable amounts of MAG, DAG, and particularly FFA which concentrate in mixed micelles as early as 4 h. Moreover, extending the incubation time led to a significant increase in TAG content, thus confirming the trend observed from 4 h to 24 h in Fig. 2. Variations in chain length and degree of unsaturation of the Tween surfactant tails modify this preferred curvature, packing rigidity and hydrocarbon chain fluidity (Israelachvili, 2011). Micelles typically incorporate guest molecules until the spontaneous curvature of the surfactant monolayer is reached, corresponding to the minimum free energy state of the system, as bending stresses are thereby minimized. Increasing the carbon chain length from C12 (Tween 20) to C18 (Tween 60) decreases membrane fluidity and increases the critical packing parameter, which may also influence micelle shape. In contrast, the introduction of a cis double bond in the C18 chain (Tween 80) creates a structural kink that disrupts tight packing, resulting in more dynamic and flexible micelles (Szymczyk et al., 2018). These structural differences can favor the capacity of Tw20 (and to a lesser extent Tw80) micelles to incorporate amphiphilic molecules such as DAG, MAG, and FFA, thereby facilitating the subsequent solubilization of larger and more hydrophobic molecules (e.g., TAG) leading to the formation of swollen micelles. Thus, one may suspect that the mixed micelles were essentially devoid of TAG-derived oil and instead enriched — perhaps exclusively — in these surface-active lipids, whereas the small lipid droplets (including swollen micelles) would contain both TAG and probably other lipid molecules.

In conclusion, the nature of the surfactant can significantly influence its ability to form mixed micelles with surface-active molecules present in non-stripped oil. By incorporating TAG molecules, these mixed micelles may play a key role in generating small lipid droplets, which can form or reorganize under low energy inputs - such as those occurring during emulsion storage.

### 3.2 Effect of emulsification parameters on lipid droplets size, population concentration and the droplet-to-micelle ratio in oil-in-water emulsions

**Fig. 3:**
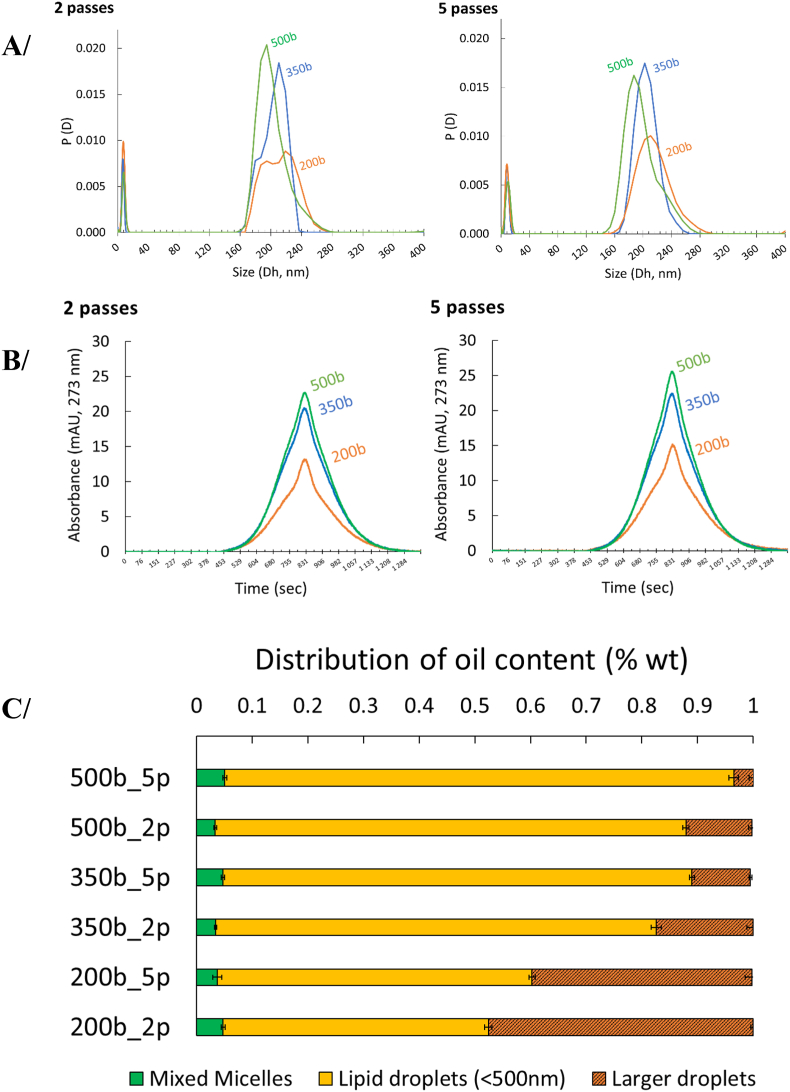
A/Hydrodynamic diameters (n = 4) of oil-in-water emulsions prepared with 1% (w/w) tung oil and 1% (w/w) Tween 20 in phosphate-buffered solution (PBS, pH 7.2), under different high-pressure homogenization conditions (200, 350, or 500 bar; 2 or 5 passes). B/Taylorgram recorded at 273 nm for the different emulsions. Analysis was conducted at 10 °C with a mobilization pressure of 50 mbar. C/SD-TDA-based estimation of oil partitioning among micelles (mixed micelles), small lipid droplets (<500 nm), and larger lipid droplets.

To investigate how the emulsification process affects droplet size and population concentrations, tung oil (1 wt%) was emulsified with Tw20 (1 wt%) in phosphate buffer (pH 7.2) under various homogenization conditions, specifically at pressures of 200, 350, and 500 bar with either 2 or 5 passes. The resulting emulsions were analyzed by SD-TDA at 273 nm to characterize lipid droplet and mixed micelle size distributions, quantify population concentrations, and evaluate the droplet-to-mixed micelle ratio. As shown in Fig. 3, SD-TDA provided detailed resolution of coexisting populations. Two main populations were detected: (i) mixed micelles ranging from ∼3 to 15 nm, and (ii) small lipid droplets ranging from ∼140 to 300 nm (note: data processing employed a maximum integration cutoff of 500 nm). Integration of Taylorgram peak areas for the different emulsions (Fig. 3B), together with the calibration curve of tung oil droplets in emulsions (Fig. 1) and mass partitioning analysis (Fig. 3A), enabled quantification of oil distribution across mixed micelles, small lipid droplets (<500 nm), and larger unresolved droplets (Fig. 3C). This latter fraction corresponds to the difference between the expected absorbance of initially added oil (1 wt%) and the quantified oil recovered in mixed micelles and small droplets. The reduced - or even completely absent - absorbance of these large lipid droplets in the Taylorgram peak may be explained by (i) creaming, which prevents their proper injection into the capillary and thus their Taylor dispersion, (ii) their size which exceeds the effective analytical range of Taylor dispersion (∼1 μm), and/or (iii) a more likely scenario, a multifactorial marked decrease in absorbed intensity combined with the detection-limit sensitivity. Interestingly, the fraction of mixed micelles remained relatively constant across all conditions (0.033 ± 0.003 to 0.051 ± 0.003 wt%), showing no significant dependence on homogenization pressure or number of passes, and closely matching the micellar solubilization capacity measured in Fig. 2 (0.061 ± 0.001 wt%). This result seems to confirm that Tw20 micelles in oil-in-water emulsions can readily incorporate surface-active lipids (DAG, MAG, FFA, tocopherols, etc.) still present in the non-stripped oil, thereby reaching a maximum level of mixed micelle formation that seems almost unaffected by the emulsification conditions. By contrast, TAG are primarily confined within lipid droplets and are more strongly influenced by energy inputs and emulsification conditions. Thus, higher pressures - and, to a lesser extent, additional passes - promote the formation of smaller droplets (<500 nm). Beyond increasing the population of small oil droplets, this also alter their relative proportion compared with the micellar population (both free micelles and mixed micelles). Therefore, the ratio of tung oil contained in droplets (<500 nm) to that solubilized in mixed micelles increased markedly with increasing homogenization pressure.

**Fig. 4:**
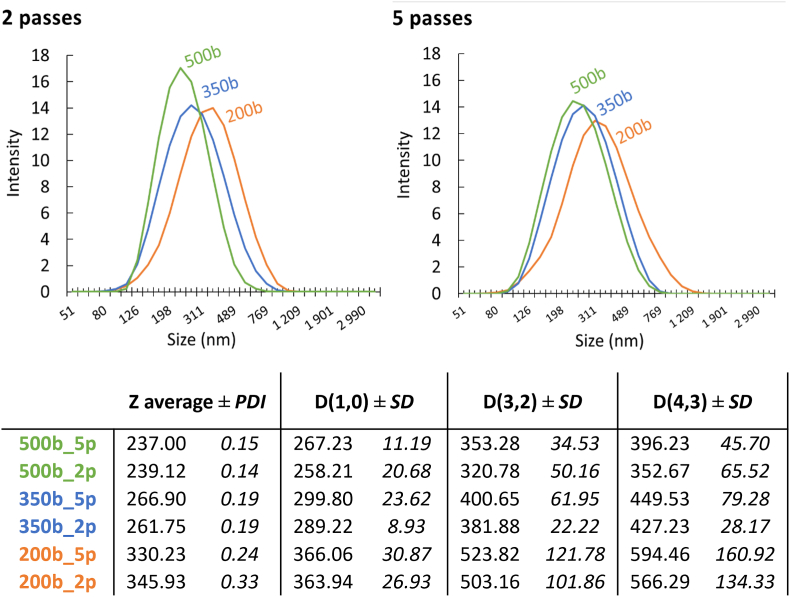
Mean DLS intensity distributions (n = 4) of oil-in-water emulsions prepared with 1% (w/w) tung oil and 1% (w/w) Tween 20 in phosphate-buffered (PBS, pH 7.2) under different high-pressure homogenization conditions (200, 350, or 500 bar; 2 or 5 passes). Reported parameters include Z-average ± PDI, D(1,0) ± SD, D(3,2) ± SD, and D(4,3) ± SD as determined from DLS measurements (n = 4).

Dynamic light scattering (DLS) was also used to evaluate the impact of emulsification parameters on lipid droplet size. This technique, based on the Brownian motion of dispersed particles and the intensity of scattered light, can suffer from limited resolution, particularly when multiple droplet populations coexist over a broad size range (from ∼5 nm to >500 nm). Nevertheless, DLS remains the most widely used and reference technique for assessing whether an oil-in-water nanoemulsion is properly dispersed and stable, for instance during oxidative stability measurements. First, as shown in Fig. 4, DLS provided more limited information compared to SD-TDA, revealing only a single, unimodal size distribution ranging from ∼100 nm to ∼1.4 μm, whereas SD-TDA resolved multiple populations. As expected, DLS was therefore unable to detect micelles (either free or mixed). At lower emulsification energies, however, DLS indicated broader droplet size distributions, reflected by increased Z-average ± PDI, D(1,0) ± SD, D(3,2) ± SD, and D(4,3) ± SD values. These findings are consistent with the SD-TDA results, which highlighted a shift of the droplet size population toward larger diameters exceeding 500 nm.

Therefore, combining SD-TDA and DLS analyses allowed the following conclusions to be drawn:

- The fraction of oil solubilized in Tw20 (mixed micelles) remained relatively constant, showing no significant dependence on homogenization pressure or number of passes, with values similar to the micellar solubilization capacity measured in the absence of high-shear emulsification.
- The oil fraction solubilized in Tw20 micelles is likely enriched (perhaps exclusively) in non-Triacylglycerol-derived molecules, such as Diacylglycerols, Monoacylglycerols, Free Fatty acids, etc. which appear to facilitate the dispersion of TAG as lipid droplets.
- The distribution of oil in smaller lipid droplets (<500 nm), and larger droplets is strongly influenced by the emulsification conditions particularly affecting the relative concentration of oil in the smallest droplet populations.
- The ratio of smaller droplets (<500 nm) to (free or mixed) micelles in oil-in-water emulsions is highly sensitive to the emulsification step, indicating that relatively high energy input is required to achieve complete redistribution of oil within the smaller droplets.

### 3.3 Investigation of the oxidative stability of Tung-oil-in-water emulsions prepared under different high-pressure homogenization conditions

**Fig. 5:**
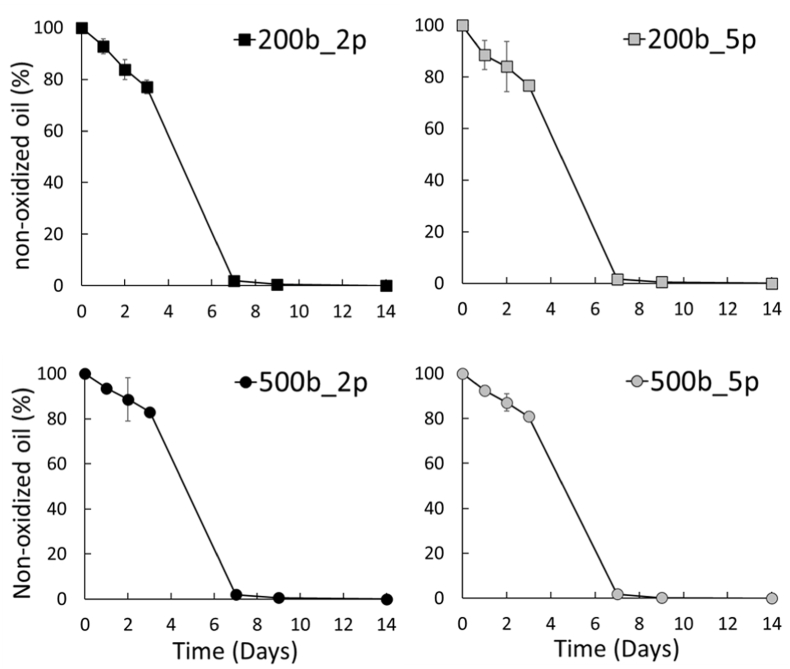
Oxidation measurement level based on Taylorgram integration peaks at 273 nm and 234 nm (n = 3) (see section 2.5) for oil-in-water emulsions containing 1% (w/w) tung oil and 1% (w/w) Tween 20 in phosphate-buffered solution (PBS, pH 7.2) prepared under different high-pressure homogenization conditions (200 and 500 bars; 2 or 5 passes). Emulsions were incubated at 30 °C for 14 days.

Due to its conjugated triene structure, eleostearic acid is highly prone to oxidation but it also exhibits a distinct absorbance pattern during the oxidation process, characterized by a simultaneous decrease at 273 nm and increase at 234 nm, connected by an isosbestic point (Laguerre et al., 2008). Therefore, in addition to resolving droplet size distributions and population concentrations, SD-TDA enables *in situ* monitoring of oxidation in Tung oil-in-water emulsions, using the equation described in section 2.8. To evaluate the influence of droplet size and population concentration on oxidative stability, 1% tung oil-in-water emulsions stabilized with Tw20 (1%wt) and homogenized at 200 and 500 bar with either 2 or 5 passes were incubated at 30 °C for 14 days (Fig. 5). Since SD-TDA cannot properly evaluate the presence of larger lipid droplets (>500 nm), the analysis focused on mixed micelles and smaller lipid droplets (<500 nm), represented by the green and orange populations, respectively (Fig. 3C).

As represented in Fig. 5, no statistically significant differences were observed in the overall oxidation kinetics of the oil. The structural diversity in the emulsion described in 3.2 was expected to result in observable differences in oxidation rates. Indeed, although the literature remains divided on whether droplet size accelerates or retards oxidation - a discrepancy largely attributed to structural variability across experimental models - we anticipated more pronounced differences. One possible explanation is that only the initial stages of lipid oxidation (e.g., <1% of total lipid oxidized) may reveal condition-dependent differences in oxidative levels, whereas our model primarily captures later stages of oxidation and may therefore lack the sensitivity required to detect such subtle variations. Another plausible explanation involves the predominance of LOO• radical addition to the conjugated double bonds of tri-unsaturated eleostearic fatty acids, which, unlike non-conjugated fatty acids, may promote polymerization and consequently limit the formation of lipid hydroperoxides (LOOH) and subsequent cleavage products which may also contribute to observed differences.

### 3.4 Investigation of oil partitioning and droplet-to-mixed micelle ratios during the oxidation of Tung-oil-in-water emulsions prepared under different high-pressure homogenization conditions

**Fig. 6:**
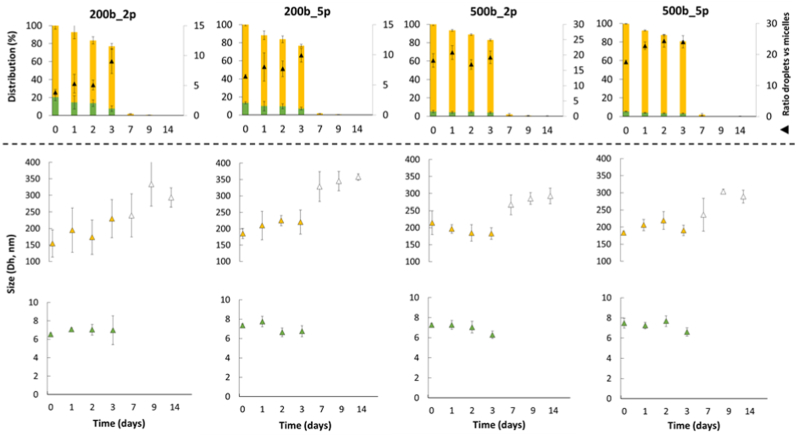
Evaluation of oil partitioning (%wt.), droplets (<500 nm) and mixed micelles size, and smaller droplet-to-mixed micelle ratios (▲) during the oxidation of 1% tung-oil-in-water emulsions stabilized with 1% Tween 20 (PBS, pH 7.2), prepared under different homogenization pressures (200 vs. 500 bar) and number of passes (2 vs. 5). The empty triangle corresponds to the size of the lipid droplet population containing fully oxidized oil, still detectable at 273 nm. Note: oxidized micelles could not be analyzed at 273 nm because their absorbance fell below the instrument's detection limit.

Fig. 6 shows the distribution of unoxidized tung oil determined from the eleostearic acid absorbance at 273 nm within micelles and smaller lipid droplets (<500 nm) over 14 days of emulsion storage, together with the size evolution of these two populations. The droplet-to–mixed micelle ratio is also shown (▲) and can be interpreted as the relative change in unoxidized tung oil distributed between these two populations during storage. As described in section 3.2, tung oil is partitioned between mixed micelles formed with Tween 20 (hydrodynamic diameter ∼7–8 nm), a population of small lipid droplets (∼200 nm), along with additional larger droplets (>500 nm) (Fig. 3D). Thus, the predominance of this largest population substantially affects the small droplet-to-mixed micelle ratio measured by SD-TDA (Fig. 6), which was significantly lower under milder homogenization conditions (200b_2p = 3.9 ± 0.5) compared to high-energy conditions (500b_5p = 17.5 ± 0.5). While the sizes of mixed micelles remained largely stable across conditions, the smaller lipid droplet population (<500 nm) exhibited distinct evolution trends. At time 0 and during storage, increasing the homogenization pressure and number of passes (from 200b_2p to 500b_5p) resulted in a marked reduction in size dispersion, with a narrower and less heterogeneous size distribution under stronger emulsification conditions. Note that the oil distribution is expressed on an eleostearic acid mass basis. Consequently, the previously discussed heterogeneity in lipid composition - namely the potential enrichment of partial glycerides or FFA in mixed micelles and the predominance of triglycerides within lipid droplets - may lead to a slight underestimation of the oil fraction present in mixed micelles when considered on a molar basis. However, incorporating this effect into the calculations is not feasible, particularly given that structural rearrangements during storage, such as the redistribution of TAG–OOH into mixed micelles, may occur.

Under high-pressure homogenization conditions (500b_2p and 500b_5p), droplet size distributions are more homogeneous (narrow dispersity for small droplets ∼200 nm and fewer droplets >500 nm) with only minor variation during the 14 days of storage. Moreover, a similar oxidation rate, with low variability across replicates, is observed between mixed micelles and small lipid droplets. As a result, a nearly constant unoxidized small droplet-to-mixed micelle ratio during oxidation (except between 0 and 1 day at 500b_5p, which remains unexplained) is observed, suggesting that the proportion of unoxidized tung oil in these populations remains largely stable over time. In these conditions, the oxidation of these two populations appear to occur at similar rates or at rates slower than the thermodynamic exchange between ∼200 nm droplets and micelles.

**Fig. 7:**
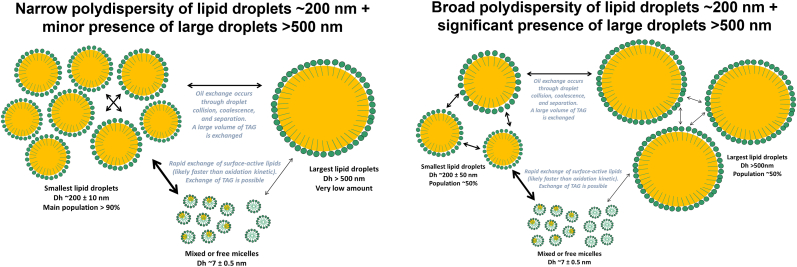
Schematic representation of non-stripped oil-in-water emulsion oxidation in the presence of Tween 20 micelles, illustrating two distinct scenarios: narrow and broad polydispersity.

Under the lowest-energy conditions (200b_2p and 200b_5p), largest variations in lipid droplet size were observed, with oxidation during storage further increasing both droplet size and heterogeneity. This behavior may be attributed to the presence of a significant fraction of large lipid droplets (>500 nm), where continuous, but slower thermodynamic equilibrium and oil exchange may occur. Under these milder emulsification conditions (broad polydispersity for small droplets (∼200 nm) and prevalence of droplets >500 nm), a slight increase in the ratio of unoxidized small lipid droplets to mixed micelles is observed during oxidation. This suggests that tung oil located within mixed micelles oxidizes more rapidly than tung oil contained in the small droplet population (<500 nm) in the presence of larger droplets (>500 nm). This would imply that the largest lipid droplets oxidize more slowly than both the small droplets and the mixed micelles. Although these differences do not significantly alter the oxidation kinetics (see section 3.3 for discussion), the presence of larger lipid droplets (>500 nm) appears to affect the oxidation behavior of the emulsions. This could be due to kinetics and volume of material exchange, as exchange between mixed micelles and small lipid droplets is rapid but limited in volume, whereas inter lipid droplets exchange is slower yet involves a larger volume (Fig. 7).

Thus, these results would suggest that the very large lipid droplets (>500 nm) oxidize more slowly, potentially acting as “reservoirs” of unoxidized TAG. This pool of TAG may redistribute to smaller droplets through inter-droplet collisions, possibly increasing the unoxidized and smaller lipid droplet-to-micelle ratio as oxidation progresses. In addition, in our emulsion, the thermodynamic equilibrium of oil between mixed micelles and small lipid droplets is rapid and dominates over any independent oxidation occurring within these sub-populations. Given the heterogeneous dispersion of eleostearic-derived lipid molecules among these populations, we were not able to observe a significant difference between the oxidation rate of TAG - primarily concentrated in the lipid droplets - and that of DAG, MAG, and FFA, which are concentrated in the mixed micelles. This lack of observable difference could also be due to the fact that, even if lipids within mixed micelles oxidize more rapidly, this effect may be counterbalanced by the redistribution of “mono”-oxidized TAG species (TAG–OOH) into the micelles or by the capacity of oxidize species to form their own micellar structures at more advanced stage of oxidation (Villeneuve et al., 2021; Hennebelle et al., 2024; Budilarto and Kamal-Eldin, 2015). Such redistribution or own micellization of oxidized species could re-establish the apparent balance of unoxidized eleostearic moieties between the droplets and the mixed micelles (ten Klooster et al., 2023b). The pronounced difference in composition between mixed micelles (rich in surface-active lipids and nearly lacking unoxidized TAG) and lipid droplets represents a key aspect for better understanding the variability often reported in the literature concerning lipid oxidation in emulsions. The quality of non-stripped oil may lead to a heterogeneous distribution of oxidizable fatty acids, depending on whether they are concentrated in micelles (DAG, MAG, FFA) or in lipid droplets (TAG). This could explain discrepancies observed between studies using different oil sources - even when derived from the same vegetable oil but differing in origin or grade - as the free fatty acid composition concentrated in mixed micelles may vary substantially. In addition, the presence of naturally occurring fat-soluble antioxidants may influence the formation of mixed micelles. This is particularly relevant for tocopherols, whose isomeric forms - strongly dependent on the plant origin of the oil - exhibit markedly different capacities to interact with the oil-water interface (Villalaín, 2025). In the same way, in studies employing stripped oils, differences in oxidative stability should not be attributed solely to the absence of endogenous antioxidants (e.g., tocopherols) but also to the removal of such surface-active lipids that would otherwise preferentially partition into mixed-micelles. Moreover, several studies have clearly demonstrated the formation of small nano-objects (∼20–30 nm), most likely corresponding to swollen micelles, in Tween-stabilized oil-in-water emulsions (ten Klooster et al., 2023a; Awad et al., 2018). However, these investigations were performed using oils such as medium-chain triglycerides or stripped rapeseed oil, which are likely depleted of endogenous surface-active lipids, thereby partially or completely eliminating their potential influence. Under such conditions, the formation of these small lipid droplets is more likely governed by the applied energy input and shear rate during emulsification, rather than by low-energy self-assembly processes involving mixed micelles formed between Tween surfactants and residual surface-active lipids naturally present in the oils. Thus, the predominance of kinetically driven oxidation pathways - already suggested in the literature to be influenced by the presence of micelles - may also need to be reconsidered in light of whether mixed micelles composed of surface-active lipids are present. All these factors could therefore influence both the triggering of oxidation (initiation step) and the antioxidant or pro-oxidant interactions between co-localized molecules (propagation step).

## 4 Conclusion

This study focused on the role of mixed micelles and lipid droplets in the oxidative stability of oil-in-water emulsions using size distribution Taylor dispersion analysis. Tween surfactants enable the formation of mixed micelles with surface-active components in non-stripped oils, whereas stripping the oil prevents the formation of such structures. This indicates that mixed micelles are likely enriched in non-TAG-derived species and other surface-active lipids such as DAG, MAG, FFA, etc. Moreover, Tween 20 - and to a lesser extent Tween 80 - promotes the incorporation of TAG molecules into mixed micelles, which can subsequently reorganize into small lipid droplets (<100 nm) under low-energy conditions. The fraction of oil solubilized in Tween 20 showed minimal dependence on homogenization pressure or number of passes, confirming that mixed micelles can form under mild energy inputs. However, the distribution of oil between small (<500 nm) and larger droplets is strongly dependent on emulsification conditions, particularly affecting the relative concentration of oil in the smallest droplets. The ratio of small droplets to free or mixed micelles in oil-in-water emulsions is highly sensitive to the energy input during emulsification, indicating that high energy is necessary for complete redistribution of oil into the smallest droplet populations. During oxidation, systems with narrow droplet size distributions and minimal large droplets maintain both the size of the smallest droplets and the proportion of non-oxidized oil. In contrast, systems with broad polydispersity and substantial larger droplets exhibit a slight increase in the fraction of non-oxidized oil in small droplets relative to mixed micelles, accompanied by growth of the smallest droplets. This behavior could be attributed to the kinetics and volume of material exchange: exchange between surfactant micelles and lipid droplets is rapid but limited in volume, whereas inter-droplet exchange is slower yet involves larger volumes. These findings highlight the critical role of surfactant type to influence mixed micelles typology, droplet size distribution, and emulsification energy in controlling oil partitioning, micelle–droplet ratios, and oxidation behavior in oil-in-water emulsions.

## CRediT authorship contribution statement

Erwann DURAND: Conceptualization, Methodology, Investigation, Funding acquisition, Formal analysis, Supervision, Validation, Writing – original draft, Writing – review & editing. Théo TRONCHO: Conceptualization, Methodology, Investigation, Formal analysis, Validation, Writing – review & editing. Hiteshree KOLI: Conceptualization, Methodology, Investigation, Formal analysis, Validation, Writing – review & editing. Camille ROBICHON: Conceptualization, Methodology, Investigation, Formal analysis, Validation, Writing – review & editing. Pierre VILLENEUVE: Conceptualization, Methodology, Investigation, Formal analysis, Validation, Writing – review & editing.

## Funding sources

This work was supported by the French government under the France 2030 initiative, managed by the National Research Agency (ANR), under grant number “ANR-23-DIVP-0003”, project “M2ProLIV”.

## Declaration of competing interest

The authors declare that they have no known competing financial interests or personal relationships that could have appeared to influence the work reported in this paper.
