# Supplementary material for: Focus on the role of mixed micelles and lipid droplets in the oxidative stability of oil-in-water emulsions using size distribution Taylor dispersion analysis
Source: Curr Res Food Sci. 2026 Feb 28;12:101369. doi: 10.1016/j.crfs.2026.101369 (PMC12972534; doi:10.1016/j.crfs.2026.101369)
Supplement: Multimedia component 1 [file mmc1.docx]

**Supplementary information**

**
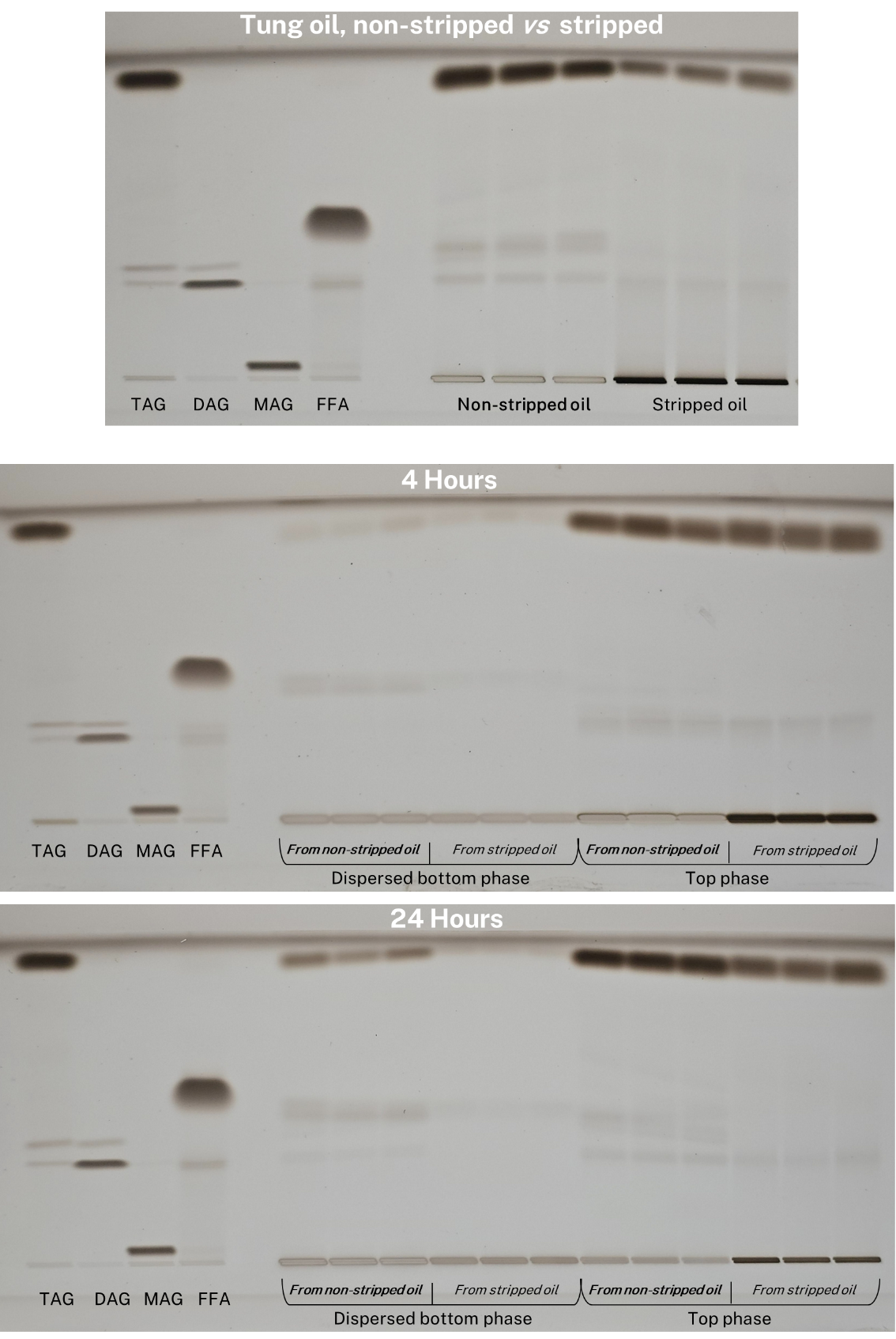
**

**Figure S1: TLC Plate revealed with copper sulphate/ phosphoric acid 85% (50/ 50 v/v)**

**Solvent: Hexane/ Diethylether/ Acetic acid: 70/30/1 (v/v/v). TAG = Triacylglycerol, DAG = Diacylglycerol, MAG = Monoacylglycerol, FFA = Free fatty acid.**
